# Confidence‐driven weighted retraining for predicting safety‐critical failures in autonomous driving systems

**DOI:** 10.1002/smr.2386

**Published:** 2021-10-05

**Authors:** Andrea Stocco, Paolo Tonella

**Affiliations:** ^1^ Software Institute USI Lugano

**Keywords:** AI testing, autonomous driving systems, continual learning, misbehavior prediction

## Abstract

Safe handling of hazardous driving situations is a task of high practical relevance for building reliable and trustworthy cyber‐physical systems such as autonomous driving systems. This task necessitates an accurate prediction system of the vehicle's confidence to prevent potentially harmful system failures on the occurrence of unpredictable conditions that make it less safe to drive. In this paper, we discuss the challenges of adapting a misbehavior predictor with knowledge mined during the execution of the main system. Then, we present a framework for the continual learning of misbehavior predictors, which records in‐field behavioral data to determine what data are appropriate for adaptation. Our framework guides adaptive retraining using a novel combination of in‐field confidence metric selection and reconstruction error‐based weighing. We evaluate our framework to improve a misbehavior predictor from the literature on the Udacity simulator for self‐driving cars. Our results show that our framework can reduce the false positive rate by a large margin and can adapt to nominal behavior drifts while maintaining the original capability to predict failures up to several seconds in advance.

## Graphical abstract

Anticipating hazardous driving situations has high practical relevance for reliable autonomous driving systems. We propose a framework for the continual adaptation of a misbehavior predictor of environmental uncertainty. Our framework guides adaptive retraining using a combination of in‐field confidence metric selection and reconstruction error‐based weighing. Our framework can reduce the false positive rate by a large margin and can adapt to nominal behavior drifts while maintaining the original capability to predict failures up to several seconds in advance.

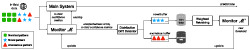

## 1 INTRODUCTION

Complex cyber‐physical systems (CPSs) are used to operate in the physical world. Nowadays, an increasing number of CPS embed one or more artificial intelligence (AI) modules, such as deep neural networks (DNNs), to perform advanced safety‐critical tasks that cannot be addressed by traditional programming.^1^, ^2^ For instance, in the context of autonomous driving, DNNs are used to predict the driving control parameters within a self‐driving car,^3^ leveraging convolutional neural networks as perception units to process digital images representing driving scenes that are used for a large variety of tasks such as lane‐keeping, pedestrian and vehicle detection. Other domains involve medical diagnosis,^4^ disease prediction,^5^ and aircraft collision avoidance.^6^

Despite the rapid advancements of AI, DNN‐based CPSs carry numerous drawbacks when exposed to open‐world operational domains. To date, DNNs are mostly tested relying on accuracy metrics on large test sets that attempt to represent a meaningful subset of the targeted operational domain. This is insufficient when the DNN is deployed within a real‐world CPS, as the in‐field inputs may differ substantially from those of the test set, thereby creating a *false notion of safety*. Indeed, erroneous predictions by the AI modules can, in turn, propagate to failures of the CPS as a whole. Incidents like those reported by Tesla,^7^ Waymo,^8^ and Uber^9^ testify situations in which the perception systems of such CPS may occasionally fail when exposed to the complexity and variety of the real world.

The main limitation of DNN‐based CPS is rooted in their lacking or limited ability to adapt to novel, ever‐changing environments. For example, DNNs are fragile to domain shifts^10^ (i.e., test data that differ from the training distribution) and data corruption^11^ (e.g., adversarial examples or sensor malfunctions) that may occur when the system is in operation. In the machine learning literature, the problem of detecting inputs that are unsupported by the model is called out‐of‐distribution detection,^12^ whereas in the software testing literature, it is mostly referred to as input validation.^13^ In either case, a popular solution to address this problem is using unsupervised anomaly detection techniques that need no a priori knowledge of the anomalies, that is, without a labeled dataset.^14^ Rather, anomaly detectors identify the data that differ from the norm and that do not belong to the nominal data distribution.

In our research, we focus on the development of monitoring techniques that keep the level of reliability and trustworthiness high when the CPS is deployed and used in production.^15^, ^16^ We used unsupervised anomaly detection to build an *anticipatory testing framework* ^15^ that equips the real‐world CPS (specifically, a self‐driving car) with a misbehavior predictor. Such a component analyzes the environment perceived via sensors, to recognize out‐of‐distribution conditions that may possibly lead to a failure.

In our previous work,^16^ of which this article is an extension, we discussed some of the challenges to building a monitoring system that learns continuously from data gathered during the operation of the main driving component. We proposed an *end‐to‐end misbehavior predictor* that continually learns how to recognize nominal cases from in‐field data collected as the system executes.^16^ Even when exposed to evolving environmental conditions, in safety‐critical settings, the true alarm rate must be very close to 1 because unsafe executions should be eliminated or reduced to a negligible probability. However, misbehavior predictors can achieve a high true alarm rate only at the price of accepting a high false alarm rate that can occur also in nominal or nearly nominal conditions, causing much driver's discomfort and negatively affecting the driving experience. We analyze the underlying causes behind a high false alarm rate, with the main cause being the class imbalance affecting the training set. In our previous work, we made misbehavior predictors more robust by leveraging data collected in the field and rebalancing the nominal data available for retraining to accommodate incoming data distribution drifts while reducing the number of false positives.

The key idea is that an invaluable opportunity for continually learning from the field is offered by the availability of a high amount of unlabeled nominal data. Although such data are useless for retraining the main driving component, because it is unlabeled, it can be still used to improve the misbehavior predictor, because no labels are required for its training. The main challenge consists of carefully selecting the nominal inputs that can be used for adaptation. Our framework automates this task by comparing the score of the misbehavior predictor with an in‐field driving quality metric. If the misbehavior predictor raises a false alarm (i.e., the driving scenario is unseen, but the driving component is indeed confident), our framework stores them in a buffer for adaptation, which is then used for retraining. However, rebalancing the training set is also expected to increase the overall training cost (training set size and training time), which grows with the number of false positives. To overcome such an issue, in this paper, we propose *in‐field confidence metric selection and reconstruction error‐based weighted retraining*. By leveraging the inner learning capabilities of DNNs and the knowledge fetched during the simulation of the autonomous driving system, we show that our technique allows a misbehavior predictor to reduce the effect of false positives while maintaining high prediction scores.

This article is a revised and expanded version of our workshop paper.^16^ We provide details on the differences between our prior work,^15^ our workshop paper,^16^ and this article.

Our prior work^15^ proposed the use of autoencoders (AEs) to estimate the black‐box confidence of DNN‐based autopilots. The results of the study highlighted a non‐negligible percentage of false positives due to the inability of AEs to correctly model the training set data distribution. In our workshop paper,^16^ we made a step ahead in understanding the root cause of such problem, which was identified as being the class imbalance in the dataset. We have also proposed a solution based on in‐field confidence metrics to automatically identify such false positives during the simulation and remove them via rebalancing the dataset prior to retraining. In terms of *conceptual contributions* of this article, we address two problems affecting the technique proposed in our previous paper,^16^ namely, exceeding training size and catastrophic forgetting (CF). In this paper, we propose confidence‐based weighted retraining (CWR) that requires no additional training data and mitigates the effects of CF. In terms of *technical contributions*, in our workshop paper,^16^ we implemented two confidence metrics, namely, the cross‐track error (CTE) and predictive uncertainty (Monte Carlo [MC]‐Dropout), on one out of three tracks of the Udacity simulator (Lake). In the current article, we allowed for a more controllable injection of the intensity of the weather conditions, which are now parameterized instead of being static as in previous works.^15^, ^16^ Moreover, we extended the computation of the confidence metrics to all three available tracks (nine scenes in total, three for training, and six for testing) and displayed the information in a new revised GUI.

The main contributions of our work are as follows:

- A framework for the continual learning of DNN‐based misbehavior predictors. We propose to update such predictors in presence of data distribution shifts using a novel combination of in‐field confidence metric selection and reconstruction error‐based weighted retraining.
- An extension of the Udacity simulator that computes in‐field confidence metrics automatically during a simulation.
- An instantiation and evaluation of our framework to improve an existing misbehavior predictor^15^ under a diverse set of in‐ and out‐of‐distribution datasets. We show that the combination yields a reduction of the false alarm rate by a large margin, without affecting the failure predictive capability, that is, the true alarm rate, thereby resulting in higher prediction effectiveness.

## 2 BACKGROUND

### 2.1 Self‐driving car case study

We exemplify our framework on the Udacity simulator for self‐driving cars.^15^ The simulator supports training and testing of an autonomous driving system that performs *behavioral cloning*; that is, the autopilot learns the *lane keeping* functionality from a dataset of driving scenes collected from a human driver.

Three driving scenes are available (Lake, Jungle, and Mountain), representing closed‐loop tracks equipped with a single vehicle with the full availability of the road section.

The simulator also allows injecting controllable operational conditions, such as weather changes (rain, snow, fog), that we will use to produce test datasets having both supported and unsupported inputs.

### 2.2 Anomalies in self‐driving cars

An *anomaly* is an observation that significantly deviates from other observations so as to arouse suspicion that it was generated by a different mechanism.^17^ Anomalies can be caused by errors in data, but they can be also indicative of new, previously unknown, underlying scenarios. A common leitmotif in anomaly detection is that anomalies are rare, unknown, and possibly diverse in nature. Thus, it is not possible to collect a labeled dataset representative of all possible anomalies.

In the context of this paper, we consider as anomalies instances of driving scenes for which a self‐driving car was not previously trained. However, if we were to select as anomalies driving scenes that are totally or quite different from the ones present in the training set, the problem would be largely oversimplified. For instance, if images of urban driving scenes are regarded as nominal and images of highways are regarded as anomalous, the problem of finding a boundary between the distributions obtained with these two sets of images becomes quite trivial.

**FIGURE 1:**
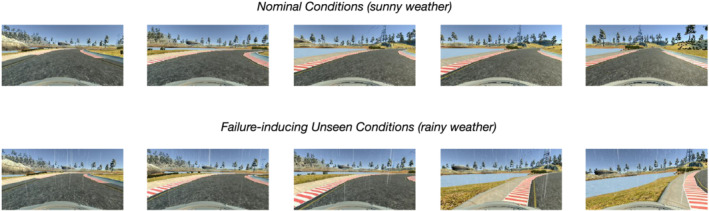
An example of the failure of the self‐driving model DAVE‐2^3^ on Udacity's Lake track (best viewed on a high resolution color monitor). (Top) In nominal driving conditions (sunny), DAVE‐2 is able to face the right bend at the default speed of 30 mph. (Bottom) Under unseen conditions (rain), DAVE‐2 fails to drive the bend at the default speed of 30 mph, and the sequence ends with a system failure (i.e., an out of track event)

A more challenging problem, which is the one faced in this paper, consists of finding such a boundary when limited perturbations to the training set driving scenes are applied. While a robust self‐driving car model should generalize well even in the presence of some minor level of perturbation, increasing perturbation levels is expected to cause a system failure (see Figure 1). The sequential nature of the driving task makes it possible that a sequence of inaccurate predictions (due to small perturbations) could ultimately lead to a failure, because of the cumulative prediction errors. By failure, we mean any deviation from the main system's requirements (e.g., lane‐keeping), such as collisions or out‐of‐track events. According to the U.S. Department of Transportation, National Highway Traffic Safety Administration (NHTSA), this kind of failures is second in frequency and first in cost among the light‐vehicle precrash scenarios by economic cost with an impact of more than 15B USD.^18^

With the goal of preventing such system‐level failures, DNN model‐level testing^1^ (i.e., exposing errors of individual predictions made by the DNN model) is not an option, because it is impossible to precisely observe and identify the causal chain going from individual, possibly small, prediction errors to failures. Offline, DNN model‐level testing techniques tend to overestimate the testing effectiveness because the model is not tested as part of a system that could compensate for small, individual errors. Thus, when testing DNNs that perform the task of driving, online testing is a fundamental step. However, in‐field online testing is very expensive and has severe limitations. Hence, it is usually preceded by extensive online testing performed within a simulation platform in which it is possible to measure, analyze, and reproduce driving failures (in the rest of the paper, we use the terms misbehavior and failure interchangeably, to refer to a deviation from the main system requirements).

### 2.3 Monitors for self‐driving car misbehavior prediction

The overall goal of this research is to build a mechanism that prevents the occurrence of system failures with high accuracy. An accurate prediction of hazardous situations is a necessary prerequisite for the implementation of a fail‐safe system having a redundant component (a *monitor*) that analyzes the data fed to the main system and, in the face of unsupported inputs, warns it to trigger countermeasures, such as recovering the system to a safe state. The main reason for having such redundancy is that the monitor can be made substantially simpler than the main system, being only focused on the task at hand, for example, misbehavior prediction. This kind of redundant architecture is quite common in practice. For instance, Tesla's autopilot system employs 48 DNNs that, together, output 1000 distinct predictions at each time step about the road layout, the surrounding infrastructure, and 3D objects found in the driving scene. *

**FIGURE 2:**
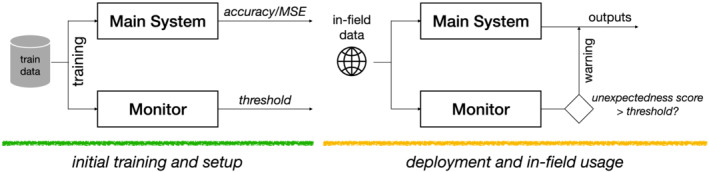
Training and usage of a static deep neural network (DNN)‐based monitor

In this paper, we consider *black‐box* environment monitors, that is, monitors that analyze the main system's input space (with no knowledge of its internal behavior) and assign an unexpectedness score, which should be low and below the threshold if such inputs are known/supported or high and above the threshold otherwise (Figure 2). The functioning of black‐box environment monitors is as follows.

First, the monitor is trained on nominal data to learn a model of normality. If the main system is also learning‐based, it is advised to use the same training set used to train the main system. Then, by selecting the accepted false alarm rate, we can determine a fixed initial threshold for deployment.^15^ The monitor is then used in the field to warn the main system if the inputs are regarded as unsupported.

### 2.4 AE‐based monitors

#### 2.4.1 Autoencoders

In many real‐world domains, such as autonomous driving, abnormal data represent rare and unexpected events for which no prior knowledge, or label, is available (i.e., the context is *unsupervised*). In the spectrum of black‐box unsupervised anomaly detection solutions, architectures based on AEs have emerged as a popular and very effective technique.^14^

AEs are DNNs whose purpose is to reconstruct the input they are given. Typically, AEs consist of two connected components—an encoder and a decoder—that are mirrored. The hidden layer encodes a given input $x\in R^{D}$ to an internal representation $z\in R^{Z}$ using a function f(x)=z. Usually *Z* ≪ *D*, where *Z* represents the dimension of the encoded representation and *D* is the dimension of the input.^15^ The decoder layer decodes the encoded representation with a reconstruction function $g(z)=x^{\prime }$, where $x^{\prime }$ is the reconstructed input x. The AE is designed to minimize a loss function L(x,g(f(x))), which measures the distance between the original data and its low‐dimensional code representation. A popular loss function for images is the mean squared error (MSE).

An interesting AE architecture is the variational autoencoder^19^ (VAE) that models the relationship between the latent variable z and the input variable x by learning the underlying probability distribution of observations using variational inference. The MSE loss function can be used for the VAE as well. However, a more suitable choice for VAEs is to use a loss function consisting of two terms: (1) the expected value of the negative log‐probability of the input x given the code z; (2) the Kullback–Leibler divergence between the distribution of z given x and the distribution of z alone. The first term plays the role of the reconstruction loss reduction because it maximizes the probability of getting the input from the code. The second term forces the VAE to minimize information loss in the latent space, hence avoiding that similar inputs are mapped to distant regions of the latent space. In the remaining of the paper, we refer to such loss function as VAE loss.

#### 2.4.2 AE reconstruction error as inference‐time unexpectedness score

In this work, we adopt AEs mainly for two reasons: (1) Their training requires no label as the input image already represents the desired output, and (2) the latent variable z ensures approximate reconstruction even in the presence of out‐of‐distribution inputs.

In our case of recognizing anomalous driving images, AEs are trained only on data with nominal instances, thus learning how to accurately reproduce the most frequent input characteristics. When facing previously unseen samples, the model will experience a worse reconstruction performance.^19^

When trained on nominal driving images, AEs learn how to reconstruct the nominal data patterns with high fidelity, whereas they worsen their reconstruction capability when unknown inputs are given (i.e., unseen driving images, hereafter referred to as anomalies). Hence, nominal and anomalous inputs are distinguished by selecting an appropriate threshold based on the reconstruction errors obtained on a validation set. Classical choices for the threshold are the maximum reconstruction error $\theta_{max}=max_{x\in X}L(x,x^{\prime })$ or a large percentile (such as 95%) $\theta_{0.95}=p_{0.95}(L(x,x^{\prime }|x\in X)$.

In this paper, we use the reconstruction error of AEs as *unexpectedness score of the driving scene*. This choice is driven by a number of reasons. First, the camera image is the main functional locus of a self‐driving car, as the majority of the vehicle's actuators are inferred just by analyzing the images retrieved by the camera through real‐time processing. Thus, AEs use information that is readily available, requiring no modifications to the main system. Second, the differences in reconstruction errors between nominal and anomalous images allow accurate differentiation. Third, the training process for AEs is quite straightforward and efficient. Even with large datasets, AEs are able to learn meaningful latent spaces even from a few examples unsupervisedly. Last, AEs are extremely fast at making predictions once trained, which makes them suitable as runtime in‐field monitoring techniques.^15^

## 3 CHALLENGES OF ADAPTATION

### 3.1 The need for adaptation

**FIGURE 3:**
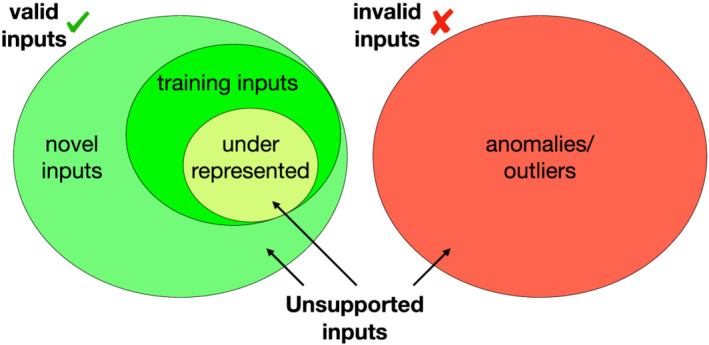
Classification of unsupported inputs

The monitor's misbehavior prediction capabilities must be updated as new knowledge becomes available. However, for nontrivial operational domains, it is virtually impossible to account for all possible nominal scenarios at training time (Figure 3). Moreover, AEs are limited in efficiently incorporating *new* knowledge after they have been trained. Finally, the thresholds used for anomaly detection are also determined offline before AEs operate in the field, which is nonoptimal when the monitor operates in a constantly changing environment.

Next, we enumerate some of the challenges that need to be addressed when designing online self‐adaptive monitors. (1) **Effectiveness**, that is, what novel data should be considered for adaptation. Ideally, the false positive rate should be kept to a tunable amount, depending on the system's or stakeholders requirements. (2) **Performance**, that is, when it is appropriate to retrain the monitor. Short‐term changes of the data distribution should not cause many false alarms or frequent model updates, while permanent context drifts should trigger model updates as soon as possible. (3) **Dependability**, that is, how to safely update the old monitor with the new one while guaranteeing service continuity. When knowledge of anomalies and novel data accumulates, the monitor needs to be updated without affecting its main functionality. (4) **Revalidation**, that is, how to regression test the new monitor that should continue to meet its requirements, hence mitigating CF.

In this work, we address the first challenge (Effectiveness) and propose and evaluate metrics that can help automate the decision process of determining what data to consider for retraining. The main advantage of using AEs is that *no labeling is required for the newly collected data*. However, the main assumption is that all training samples are reconstructed equally well. Training optimal AEs for misbehavior prediction is indeed a difficult endeavor. Large latent spaces may allow generalization to patterns of anomalous images, thereby increasing the number of false negatives because also anomalous images are reconstructed well. On the other hand, small‐sized latent spaces may affect the reconstruction of nominal images, resulting in many false positives.

Moreover, when used in production, the observed data may differ from those used for training. There could be cases in which the main system generalizes better than the monitor and then false alarms would be *mistakenly* reported. On the other hand, if the monitor generalizes too much to hazardous scenarios that are unsupported by the main system, true alarms could be *missed*.

### 3.2 Classes of unsupported inputs

A major drawback of DNNs (and thus, AEs) consists of their inability to automatically discern supported (valid) from unsupported (invalid) inputs. In fact, given an input vector, a DNN will produce an output even if the input is meaningless in the domain where the DNN is supposed to operate.^20^ Thus, monitors are used to avoiding DNNs processing unsupported inputs, which may cause unpredictable predictions, potentially causing system‐level failures, if not handled properly.

Figure 3 categorizes them into three classes based on their proximity to the training set data distribution. The figure highlights the complexity and the variety of scenarios that AE monitors should aim at.

Supported inputs pertain to the same data distribution of the ones used for training (*in‐distribution inputs*), and therefore, they *should* be handled correctly by the DNN.

Unsupported inputs, on the other hand, pertain to a different data distribution than the one used for training (*out‐of‐distribution inputs*). Underrepresented inputs are the first category of inputs that can cause a failure of the monitor, due to their low occurrences, as compared with other, more represented, classes.

A second category consists of *novel data*, that is, data in the domain of validity of what the DNN should support, but that are not yet represented *at all* in the training set and should therefore be added to it as representative of new classes of data, when available. This category is the main target of continual learning and adaptation. Finally, the third category of unsupported inputs that can be found in real‐world domains consists of *anomalous data*, that is, inputs that are not in the validity domain, and should therefore be recognized and discarded.

## 4 CONFIDENCE‐BASED CONTINUAL ONLINE MONITORING THROUGH WEIGHTED RETRAINING

The goal of our approach is to adapt an existing monitor M that uses a static threshold for misbehavior prediction (see Figure 2) by equipping it with *online learning* capability and incremental model updating strategies. Our idea revolves around an interplay between the main system and the monitor. By establishing a perpetual information exchange cycle, we collect in‐field confidence indicators of the main system's behavior to guide the improvement of the monitor.

Our framework leverages two opportunities. First, it uses in‐field behavioral confidence indicators to refine the definition of positive and negative cases. Second, it collects and relies on data *that require no label* as AE‐based monitors are trained with no supervision.

More specifically, the detection of positives (i.e., samples whose reconstruction errors are above the threshold) and negatives (i.e., negative samples whose reconstruction errors are below the threshold) is based only on a black‐box analysis of the sole inputs, which is immaterial to how the system is actually behaving in the field in response to such inputs. In the absence of ground truth, we hybridize the definition of positive and negative cases for misbehavior prediction by taking into account the main system's behavior. The observed samples are regarded as empirically valid, because they have been collected during the nominal execution of the system, as far as the feedback from the system is correct (i.e., no safety oracle is violated^21^). It is important to highlight that our focus is on updating and improving the monitor and not the main system.

If, during the in‐field learning phase, there is evidence that the faced data distribution shift is too large to be managed correctly by the main system (i.e., safety oracles are repeatedly violated), then in‐field monitoring is no longer an option, and the main system has to be shut down for additional offline retraining and testing. The collected data can be used for such retraining (at the price of manual labeling operations) or to produce test cases that mimic the novel conditions found in the field.^20^

### 4.1 Approach

**FIGURE 4:**
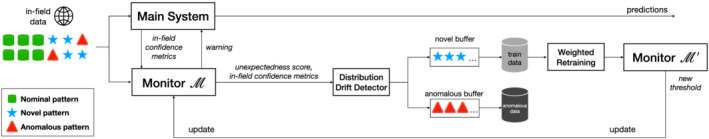
Our monitoring framework for adaptive misbehavior prediction with in‐field confidence‐driven weighted retraining

Figure 4 illustrates the usage scenario of our framework. Once the monitor is initialized (e.g., after the initial training), it is ready for online misbehavior prediction. The monitor predicts the unexpectedness scores and receives runtime behavior indicators data as the main system executes. The main thread works on real‐time misbehavior prediction, whereas a secondary thread (Distribution Drift Detector) collects behavioral in‐field data from the main system continuously that are later used for adaptation (Weighted Retraining). Next, we describe these two components in detail.

#### 4.1.1 Distribution drift detector with in‐field confidence metrics

The distribution drift detector determines what data to store for adaptation. It uses in‐field confidence metrics to understand whether the samples unsupported by M are also unsupported by the main system. On the occurrence of alarms raised by the monitor or when confidence metrics indicate poor system performance, anomalous data are stored into an anomaly buffer for later evaluation. When confidence metrics indicate acceptable system performance, but data are classified as novel, the collected data are added to the training set of the monitor M, which is ready for retraining: The sample is stored in a buffer containing novel and possibly mistakenly predicted nominal data. A new threshold is computed, and the new monitor $M^{\prime }$ substitutes the old one.

As required by our framework, we need an automated way to determine confidence metrics for the main system. We implemented two in‐field metrics that can be used to assess the quality of driving, namely, *predictive uncertainty* and *lateral deviation*. The former is a white‐box confidence metric for DNNs, whereas the latter is a black‐box confidence measure of the whole autonomous vehicle behavior. In our study, we are interested to assess whether such metrics can be used to guide the retraining of a better monitor, whether one metric is preferable over the other, along with their benefits and limitations.

##### Predictive uncertainty

The first considered metric is an internal measure of the system's confidence in its own predictions. We use the predictive variance of dropout‐based DNNs called MC dropout, which we will refer hereafter simply to as MC‐Dropout.^22^

**FIGURE 5:**
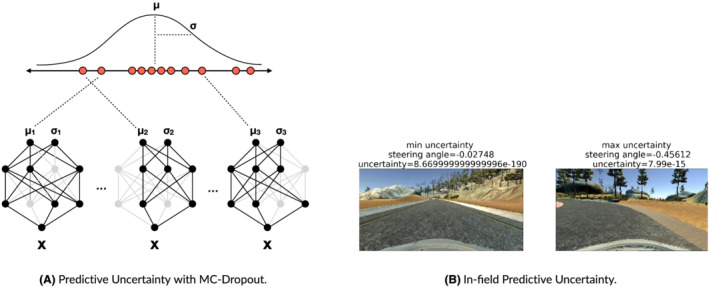
Graphical illustration of the predictive uncertainty using Monte Carlo (MC)‐Dropout (A). Usage for measuring the confidence of a self‐driving car deep neural network (DNN) model (B). The frames having the minimum/maximum values of MC‐Dropout predictive uncertainty for the Lake track. The final predicted steering angle corresponds to the mean *μ* of 128 stochastic passes by DAVE‐2, whereas the uncertainty value is given by the variance *σ* of such predictions. The confidence is higher on straight roads for which the steering angle prediction for DAVE‐2 is easier (lower uncertainty values), whereas the confidence is lower when facing bends at high speed (higher uncertainty values). (A) Predictive uncertainty with MC‐Dropout and (B) in‐field predictive uncertainty

In DNNs, dropout layers are used at training time as a regularization method to avoid overfitting, whereas at testing time, for efficiency reasons, dropout layers are usually disabled: All nodes and connections are kept, and weights are properly adjusted (e.g., multiplied by the *keep ratio*, defined as *1 ‐ dropout rate*). Thus, at testing time, the prediction is deterministic because, without other sources of randomness, the model will always predict the same label or value for the same test data point. On the contrary, when estimating predictive uncertainty with MC‐dropout, the dropout layers are enabled at both training and testing time. During testing, predictions are no longer deterministic, being dependent on which nodes/links are randomly chosen by the network. Therefore, given the same test data point, the model could predict slightly different values every time the point is passed through the network (see Figure 5A). Therefore, this mechanism is used to generate samples interpreted as a probability distribution (this is called Bayesian interpretation in the machine learning literature^22^). In practice, the value predicted by the DNN will be the expected value (mean) of such probability distribution. Moreover, by collecting multiple predictions for a single input, each with a different realization of weights due to dropout layers, it is possible to account for model uncertainty: The variance of the observed probability distribution quantifies such uncertainty (see Figure 5B). A higher variance marks lower confidence, whereas a lower variance indicates higher confidence. For a complete overview of MC‐Dropout, we refer the reader to the relevant literature.^22^

The rationale for using MC‐Dropout is that supported inputs are expected to be characterized by low DNN uncertainties, whereas unsupported inputs are expected to increase it (see Figure 5C). The MC‐Dropout approach circumvents the computational bottlenecks associated with having to train an ensemble of DNNs in order to estimate predictive uncertainty. MC‐Dropout provides a scalable way to estimate a predictive distribution, and it has been successfully applied to the self‐driving car domain^23^ as a measure to approximate uncertainty for DNNs that solve regression problems, such as DAVE‐2. The number of stochastic forward passes through the network should be determined empirically; Gal and Ghahramani^22^ suggest values as small as 10 for a reasonable estimation of the predictive mean and uncertainty. For the implementation of MC‐Dropout‐based DAVE‐2 model, we followed the guidelines provided in a similar experiment^23^ in which, for the MC‐Dropout predictions, a batch size of 128 was used (the same used by the DAVE‐2 model), as a good trade‐off between processing time and accuracy of the predictive distribution sampling.

##### Lateral Deviation

The second considered metric is a black‐box measure of the car's distance from the center of the road, which we refer to as *lateral deviation*.^16^, ^21^

**FIGURE 6:**
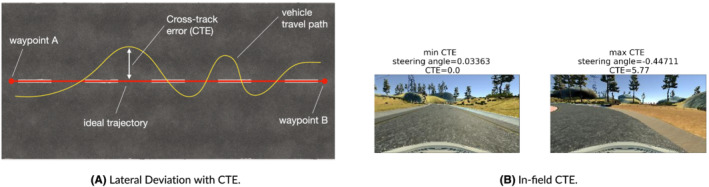
Graphical illustration of the lateral deviation using cross‐track error (CTE) (A). Usage for measuring the confidence of a self‐driving car deep neural network (DNN) model (B). The images show the frames having the minimum/maximum values of CTE for Lake track. The confidence is higher on straight roads for which the steering angle prediction for DAVE‐2 is easier (lower CTE values), whereas it is lower when facing bends at high speed (higher CTE values). (A) Lateral Deviation with CTE and (B) In‐field CTE

In a self‐driving car simulator, the DNN responsible for lane‐keeping generates a sequence of inputs for the car's actuators that define an optimal collision‐free trajectory according to the vehicle's dynamics. The car's controller then instruments the outputs of the (simulated) actuators to follow that trajectory. One way to assess the correctness of the DNN predictions is by checking whether the predictions sent to the vehicle's controller minimize the distance between the predicted position of a vehicle and the corresponding position at the reference trajectory. The error between a predicted location and the corresponding location at the reference trajectory is called the CTE, which is a good approximation of the lateral deviation (see Figure 6A).

The rationale for using lateral deviation is that unsupported inputs may cause erroneous steering angle predictions, thus lowering the chances of the self‐driving car to follow the ideal trajectory (see Figure 6B). Thus, CTE can be used as an external confidence metric of an autonomous driving system that performs lane keeping, as well as similar distance‐based tasks (e.g., assisted parking). In the training sets used in our study, the car is trained to follow the center of the road. Thus, the choice of the center of the lane is instrumental to our experimental setting, but, in principle, the very definition of CTE can be adapted to measure the distance with *any* ideal trajectory, not only the center of the lane. In fact, traveling following the center lane may not be the best trajectory that minimizes the time required to complete a full lap.

The tracks in the Udacity simulator are equipped with waypoints, i.e., phantom objects that are used to mark distinct sectors. We implemented a component within the Udacity simulator that measures the CTE as the distance from the center of the car's cruising position to the center of the road on the ideal trajectory between the planned route given by two consecutive waypoints.

##### Threshold Selection

Table 1 shows how we use the internal and confidence metrics (respectively, MC‐Dropout and CTE) to classify the collected data as likely true/false positive/negative cases (LTP, LFP, LTN, LFN), along with the thresholds used in our study. Adaptation of the misbehavior predictor makes use of the LFP data, i.e., those underrepresented or missing inputs that cause the misbehavior predictor to trigger a false alarm.

**TABLE 1:** Classification of likely true/false positive and negative cases (LTP, LFP, LTN, LFN) for misbehavior prediction based on unexpectedness, uncertainty, or lateral deviation

Metric • Threshold	Rule	Lake	Jungle	Mountain
Autoencoder Reconstruction Loss • *γ*_95_			
	LTP → *context* = *unexpected* ∧ *loss* > *γ*_95_	$\gamma_{95}=318$	$\gamma_{95}=326$	$\gamma_{95}=253$
	LTN → *context* = *nominal* ∧ *loss* < *γ*_95_			
	LFP → *context* = *nominal* ∧ *loss* > *γ*_95_			
	LFN → *context* = *unexpected* ∧ *loss* < *γ*_95_			
MC‐Dropout • *θ*_95_				
	LTP → *Unc* > *θ*_95_ ∧ *loss* > *γ*_95_	$\theta_{95}=1.04\times 10^{-15}$	$\theta_{95}=1.04\times 10^{-2}$	$\theta_{95}=4.78\times 10^{-4}$
	LTN → *Unc* < *θ*_95_ ∧ *loss* < *γ*_95_			
	LFP → *Unc* < *θ*_95_ ∧ *loss* > *γ*_95_			
	LFN → *Unc* > *θ*_95_ ∧ *loss* < *γ*_95_			
CTE • *ϵ* (mt)^a^
	LTP → *CTE* > *ϵ* ∧ *loss* > *γ*_95_	ϵ=1.5	ϵ=0.5	ϵ=1.5
	LTN → *CTE* < *ϵ* ∧ *loss* < *γ*_95_			
	LFP → *CTE* < *ϵ* ∧ *loss* > *γ*_95_			
	LFN → *CTE* > *ϵ* ∧ *loss* < *γ*_95_			

*Note*: Thresholds refer to our best performing misbehavior predictor, the VAE with latent dimension of 16 and MSE loss.

Abbreviations: CTE, cross‐track error; MC, Monte Carlo.

a Considering the width of the vehicle in the Udacity simulator and the widths of the tracks.

Concerning the AE loss, threshold *γ*_95_ was selected by estimating the shape *κ* and scale *θ* parameters of the fitted Gamma distribution of the reconstruction errors and selecting the desired accepted false alarm rate.^15^ Similar to previous works, we adopted a 95% percentile. Concerning the uncertainty values, threshold *θ*_95_ was selected with an analogous strategy. The threshold *ϵ* for the CTE is expressed in meters and was selected empirically, by observation of the geometry of the simulated car and road sizes in the Udacity simulator. Table 1 reports all thresholds used in our empirical study, on a per‐track basis.

### 4.2 Triggering adaptation and model retraining

In continual learning scenarios, a viable option to update the DNN‐based monitor is through retraining. Retraining should aim to incorporate the new knowledge without deteriorating the existing one (CF). In DNNs, this essentially means that a new set of weights obtained through retraining should handle both underrepresented and new inputs, without sacrificing the performance on old inputs.

During the online processing, if the monitor detects that the model no longer fits the current data, then model updating is triggered through retraining. The key idea of our approach consists in reducing the effect of underrepresented inputs, thereby learning a more accurate model of the normal data distribution. Likewise, our method can be also used to refine the definition of nominal class with additional novel samples. After retraining, a new threshold is also computed, by selecting the desired accepted false alarm rate on the new distribution. Finally, the new monitor $M^{\prime }$ seamlessly substitutes the monitor M.

Next, we describe two weighted retraining methods that we implemented and evaluated in our work, namely RDR and CWR.

#### 4.2.1 Rebalanced dataset retraining

In our previous paper,^16^ we instrumented retraining of the monitor by rebalancing the composition of the training set. The approach works as follows. First, likely false‐positive frames are selected through in‐field confidence metrics. Then, the majority class (i.e., the true negatives, the frames that are reconstructed well) is downsampled, to decrease its relative importance. Last, the minority class (i.e., the likely false positives) are oversampled through replication. Thus, the in‐field confidence metrics are only used for the selection of the frames to be used for adaptation. Such a method requires two parameters *d* and *o* that specify the amount of downsampling and oversampling, respectively. For instance, if the simulation time is approximately 180 s, and the simulator recorded 2700 frames, the fps is 15. Suppose that 2314 frames are regarded as false negatives. Thus, with d=2 (i.e., we retain half of the frames), 2314/2=1157 frames are retained in the training set. Conversely, for the 26 frames regarded as likely false positive, we duplicated each frame by a factor of *o*. If o=2, then each frame is added twice to the training set, giving a final training set of 52+1157=1209. Such a technique quickly increases the dimension of the training set if the false positive rate is high, even with slightly increasing values of *d*. For instance, if the false positives are 700 and *d* is set to 3, the final dataset would have size 1400+1000=3100(+15%).

Thus, the dataset weighting retraining technique is advantageous only when a low number of false positives affect the data distribution. Moreover, a proper balance between the parameters *d* and *o* may be nontrivial to find.

#### 4.2.2 Confidence‐based weighted retraining

A more elegant technique consists of weighting the samples according to their relative importance and let the neural network optimize a *weighted function*. Weighted retraining needs a set of weights that are plausible for the given domain. There are no universal rules for weight selection, except that all weights must be strictly positive values, because negative weights incentivize poor model performance, and zero weights are equivalent to discarding a sample.

In this paper, we adopt a conceptually simple solution that employs the reconstruction losses of the AE as a meaningful way to weigh each sample. The idea is that reconstruction losses are always strictly positive values and yield higher weights for poorly reconstructed samples and lower weights for good reconstructed samples. Thus, during retraining, the learned distribution would change negligibly in presence of good reconstructed samples, whereas the AE model will significantly update the training distribution to incorporate poorly reconstructed samples into the latent space. This happens because, unlike the rebalanced dataset retraining (RDR) strategy, CWR *minimizes a whole weighted loss*. Specifically, each training image $x_{i}$ is combined with a weight *w*_*i*_. Each learned weight *w*_*i*_ is a function of confidence that the model associates to the specific sample *i*. A weighted reconstruction loss is computed for the AE, notationally $L_{w}=\overset{N}{\underset{i=1}{\sum }}w_{i}x_{i},g(f(x_{i}))$. Note that weights are needed only at training time and then weights are no longer needed during validation or deployment. Moreover, no additional samples or data duplication is needed. CWR transforms the AE from a passive reconstructor into an architecture that ensures that the latent space is constantly representative of the most updated and relevant points observed in the field.

##### When to retrain

Several strategies are possible to decide when to trigger a model update, such as setting a limit to the buffer's size, or a time limit, possibly combined with the former. Our general guideline is that small distribution drifts can be rapidly incorporated; hence, a small buffer size would help achieve fast adaptation. However, a drawback is that the retraining operation is quite computationally and time‐demanding for an online setting; thus, if the buffer size is too small, there is a chance that the monitor will be consuming too many computational resources due to frequent retraining operations. A proper trade‐off should be chosen depending on the observed rate of novel samples and on the available computational resources.

## 5 EXPERIMENTAL EVALUATION

We performed an empirical study to assess the effectiveness of our framework to improve a misbehavior predictor from the literature, the VAE used within the tool SelfOracle.^15^ Improvement is assessed as the capability of the retrained misbehavior predictor at maintaining a high detection rate while keeping the false alarms low in presence of underrepresented inputs and data distribution shifts. Our experiments rely on simulation testing using the open‐source Udacity simulator for self‐driving cars used in similar testing works.^15^, ^16^, ^21^

### 5.1 Research questions and metrics

We consider the following research questions:

**RQ_1_ (LFP reduction):** *How effective is our framework in reducing the likely false positive rate occurring in nominal conditions? How much is the catastrophic forgetting induced by retraining?* The first research question aims to quantify the reduction of the false positives in nominal conditions of misbehavior predictors thanks to our approach. Moreover, we quantify the effect of the CF retraining, which should be mitigated. In fact, adaptation to the distribution shifts should not happen at the expense of higher reconstruction errors in nominal conditions.

**RQ_2_ (misbehavior prediction):** *How effective are the misbehavior predictors in predicting failures after retraining?* The second research question aims to assess whether our retraining technique does preserve the true positive rate of the retrained misbehavior predictors.

**RQ_3_ (internal validation):** *How does the misbehavior predictors effectiveness vary when considering different configurations?*

- **RQ_3.1_ (loss):** *How does the misbehavior prediction rate vary when considering different loss functions?*
- **RQ_3.2_ (latent space size):** *How does the misbehavior prediction rate vary when considering different latent space sizes?*
- **RQ_3.3_ (in‐field confidence metric):** *How does the misbehavior prediction rate vary when considering different in‐field confidence metrics?*

The third research question aims to evaluate how the internal factors of our framework affect the effectiveness of the misbehavior predictors.

### 5.2 Setup

#### 5.2.1 Datasets: Nominal and unseen conditions

We set the nominal conditions in the Udacity simulator as *sunny* weather, which is the default weather condition in each road track scene.

To generate simulations with unseen conditions that could cause major failures of the driving component, we performed simulations activating a single unexpected condition, namely, *rain*. In the Udacity simulator, we set the rain particles emission rate at fixed timestamps during the simulation. The intensity of the effect ranges from a minimum of 100 to a maximum of 10,000 particles per second so as to expose the car to increasingly extreme conditions ultimately leading to system‐level failures (Figure 1). Our choice of rain as an unsupported condition is just an experimental design choice. In the testing autonomous driving systems literature, this “leave‐one‐out” approach is fairly common. There is no special reason for this specific choice of supported/unsupported conditions and different permutations would be of course allowed (e.g., supported = night + rainy; unsupported = snowy). The only important prerequisite is that the chosen unsupported condition is unseen at training time.

#### 5.2.2 Objects of study

##### Framework's configurations

We implemented different configurations of our framework, varying, in turn, the dimension of the latent space and the loss function used to minimize the reconstruction error.

##### Baseline

We use our previous retraining technique RDR^15^ as a baseline for the novel retraining technique CWR. The value for downsampling *d* was chosen to select only 3 frames per second (fps) of simulation in the minority class (other than the default value of about 13–18 fps), and the value for oversampling *o* was set to 2. Additionally, for RQ1, we use the original VAE from the study by Stocco et al,^15^ which was the singled‐image misbehavior predictor that showed the best performance in such a related study. The VAE has a latent dimension of 2, and it is trained to minimize the MSE loss between the input and reconstructed image.

#### 5.2.3 Training details

##### Self‐driving car model

We used the existing dataset^15^ of simulated driving data to train a lane‐stable DAVE‐2^3^ self‐driving car model. For each track, the training set contains 10 laps on nominal sunny conditions following two different track orientations (normal, reverse) and additional data for recovery. For each time frame (around 13 fps), three images are collected from three front‐facing cameras, one positioned at the center of the car, one facing left, and one facing right. Each image is labeled with the ground truth steering angle value for that driving image. The maximum driving speed of the driving model was capped to 30 mph during data generation, the default value in the Udacity simulator.

For each track, we trained an individual DAVE‐2 model. This facilitated training and converged towards a robust model. The number of epochs was set to 500, with a batch size of 128 and a learning rate of 0.0001. We used early stopping with a patience of 10 and a minimum loss change of 0.0005 on the validation set. The network uses the Adam optimizer to minimize the MSE between the predicted steering angles and the ground truth value. We used data augmentation to mitigate the lack of image diversity in the training data. Specifically, 60% of the data was augmented through different image transformation techniques (e.g., flipping, translation, shadowing, and brightness). We cropped the images to 80 × 160 and converted them from RGB to YUV color space. The training was performed on a machine featuring an Nvidia GPU GeForce RTX 2060 with 6 GB of memory. This training was meant to create solid models for testing, that is, able to drive multiple laps on each track under nominal conditions without showing any misbehavior in terms of crashes or out‐of‐track events.

##### Autoencoders

For each track, and for each configuration of our framework, we trained an individual VAE model. Unlike the DAVE‐2 model, we used only the center‐facing camera, because it is the only one used by the model during the testing phase for predicting the steering angles for driving the vehicle. No data augmentation was applied to the AEs training, to avoid making them robust to image perturbations that they should instead fail to reconstruct.

As a training set, we used the same one used to train the DAVE‐2 model, with the exception of using images from one lap only for each track, which is sufficient to train a robust VAE model (i.e., finding a function to reconstruct images is way easier than finding a function between an image and a real number representing a value for a car's actuator). The number of epochs was set to 100, with a learning rate of 0.0001. The network uses the Adam optimizer to minimize either the MSE or the variational (VAE) loss. The latent space dimensions were chosen in the range [2, 4, 8, 16]. The maximum dimension for our case study was chosen empirically, during our early experiments. For the retraining phase, we limited the number of epochs to 50, because the set of weights was already initialized during the first training and fewer passes on the data were required for convergence.

### 5.3 Procedure and metrics

To answer our research questions, we performed two studies. The first study concerns the detection and reduction of class imbalance, that is, underrepresented inputs on nominal conditions that cause the misbehavior predictors to raise false alarms. The second study is related to the prediction of failures on injected novel unseen conditions. To this aim, for each track, we executed several two‐lap simulations on the Udacity simulator, for each considered condition.

#### 5.3.1 RQ_1_ (LFP reduction)

We constructed a test set performing one simulation in the same nominal conditions as the training set (i.e., sunny weather). For each frame, the simulator also recorded the in‐field behavioral metrics MC‐Dropout and CTE. Then, we used our framework to compute the reconstruction error on all images of such a test set for all misbehavior predictors and estimated the number of likely false positives (LFP or likely false alarms) in nominal conditions using the in‐field behavioral metrics MC‐Dropout and CTE, according to the ruleset and thresholds in Table 1.

**FIGURE 7:**
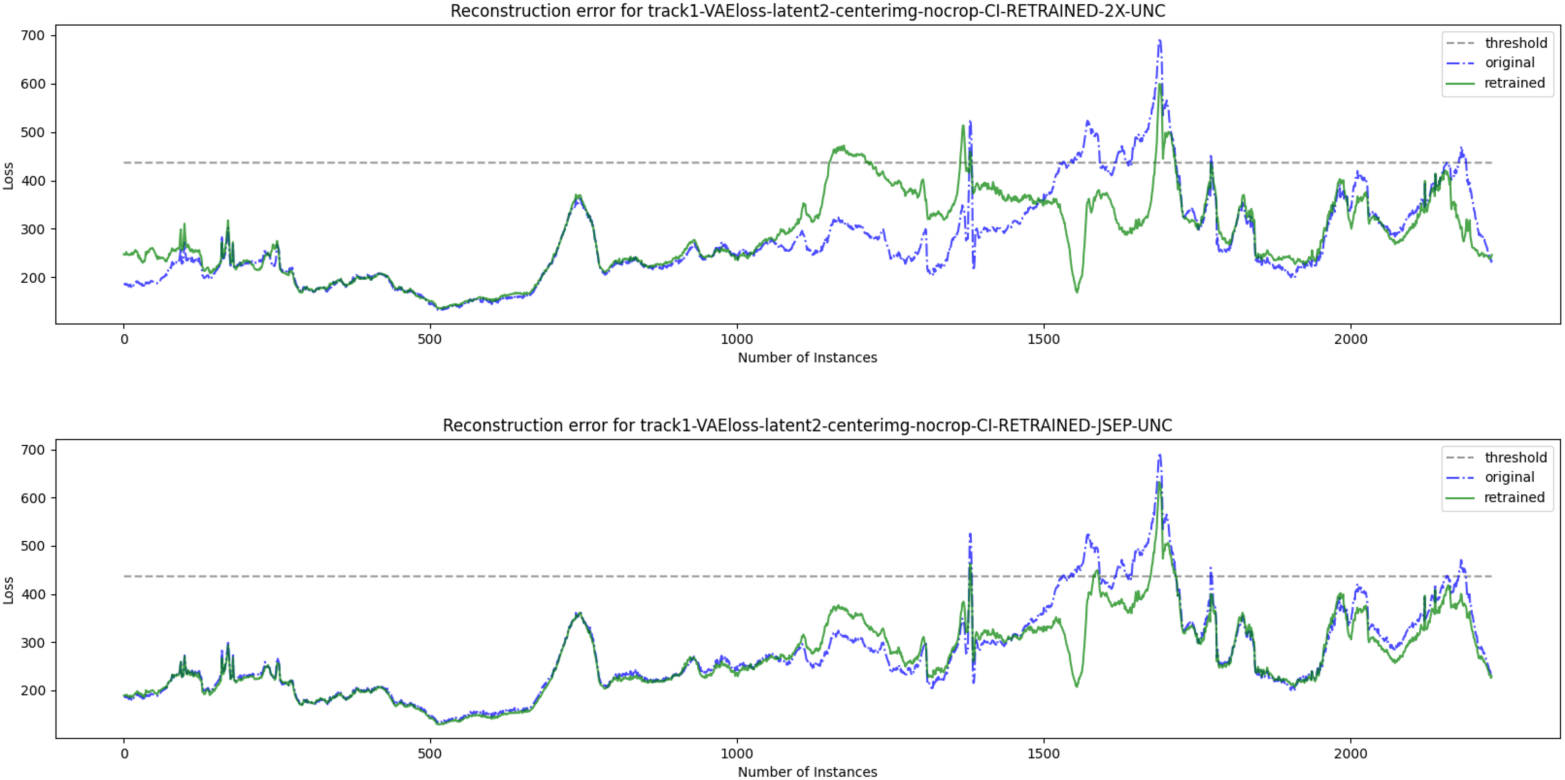
Exemplary illustration of catastrophic forgetting in autoencoders. Reconstruction errors of driving images for the Lake track, using a misbehavior predictor based on the variational autoencoder (VAE) loss and a latent dimension of 2. (Top) Results for rebalanced dataset retraining (RDR), which does reduce many false positives and causes much catastrophic forgetting, turning many true negatives to false positives (e.g., 1100–1300). (Bottom) Results for confdence‐based weighted retraining (CWR), which does reduce many false positives, but it is also less disruptive towards the original training set data distribution

After detecting the likely false positive samples, we retrained the misbehavior predictors twice, using, in turn, the RDR and CWR. Finally, we executed further simulations in nominal conditions to determine the number of false positives after retraining. To assess *effectiveness*, we measured the number of likely false positives detected in each configuration of our framework, using the threshold prior to the adaptation phase. Moreover, we quantify CF by using a custom metric $\bar{CF}$. We subtract the reconstruction errors *before* and *after* retraining only for those frames that were below the estimated threshold (i.e., our notion of being reconstructed well). Notationally, $\bar{CF}=\frac{1}{\left|L\right|}\sum_{x\in L}|\;\|x-x^{\prime }\|-\|x-x^{\prime \prime }\|\;|$, where $L=\{x|\;\|x-x^{\prime }\|<\gamma_{95}\}$ and $x^{\prime },\;x^{\prime \prime }$ are the images reconstructed by the AE for an input x before/after adaptation. Basically, we are interested in understanding whether retraining preserves or degrades the AE's effectiveness on the part of the distribution that was reconstructed well after the first training (Figure 7).

#### 5.3.2 RQ_2_ (misbehavior prediction)

We constructed a test set performing simulations on unseen conditions (i.e., rainy weather) and evaluated the misbehavior predictors in detecting the number of true positives. Following the setting by Stocco et al,^15^ we apply a time‐series analysis in the form of a simple moving average. Individual reconstruction error *e*_*t*_ at time *t* might be susceptible to single‐frame outliers (which are not expected to have a big impact on the driving of the car) but would indeed make the misbehavior predictor falsely report an anomalous context. The simple moving average computes the arithmetic mean of reconstruction errors over a moving window *w* containing *k* frames. We have chosen a window size for *w* that corresponds to 1 s of simulation in the Udacity simulator, by dividing the number of frames retrieved during a simulation by the simulation time. For instance, if the simulator recorded 2340 frames in 180 s, the fps is 13; hence, w=13.

The simulator automatically labels individual frames in which failures occur (i.e., crashes or out‐of‐track events) as anomalous. Because our framework is expected to *predict* misbehaviors ahead of time, we labeled the frames preceding the misbehavior as anomalous as well (*pre‐failure* sequences). The frames in which failure occurs are discarded. For *RQ*_2_, we also do not need to consider the nominal frames. On the list of pre‐failure sequences, we compute the true positive rate (i.e., the number of correct misbehavior predictions) and the false negative rate (i.e., the number of missed misbehavior predictions) by our framework at a different time to misbehavior (TTM), that is, considering different detection and reaction windows sizes, in the range [1, 2, 3, 4, 5]. If the average loss score for the images in that window is higher than the automatically estimated threshold *γ*_95_, our framework triggers an alarm. Consequently, a true positive is defined when our framework triggers an alarm during such anomalous windows during the specified TTM. Conversely, a false negative occurs when our framework does not trigger an alarm during an anomalous window, thus failing at predicting misbehavior.

To fully assess *effectiveness*, the false positive and true negative rates were measured in nominal condition simulations to which analogous windowing was applied. In both settings, we used the threshold prior to the adaptation phase.

Our goal is to achieve high recall or true positive rate (TPR, defined as TP/[TP + FN]), that is, true alarms while minimizing the complement of specificity, or false positive rate (FPR, defined as FP/[TN + FP]), that is, labeling safe situations as unsafe. We are also interested in the F1‐score $\left(F_{1}=2\cdot \text{Precision}\times \text{Recall}/\text{Precision}+\text{Recall}\right)$ because a high F1‐score at a given threshold can be reached only when both precision and recall are high. We also consider two threshold‐independent metrics for evaluating classifiers at various thresholds: AUC‐ROC (area under the curve of the receiver operating characteristics) and AUC‐PRC (area under the precision–recall curve). We included AUC‐PRC because AUC‐ROC is not informative when data are heavily unbalanced, because unseen situations are supposed to be rare as opposed to nominal ones. We recall that in unseen conditions, the ideal situation for a misbehavior predictor would be to have TPR = 1 and FNR = 0.

#### 5.3.3 RQ_3_ (internal validation)

To answer RQ_3_, we compare the different metrics for RQ_1_ (LFP reduction and CF) and for RQ_2_ (Precision, Recall, AUC‐ROC, and AUC‐PRC) across misbehavior predictor configurations (i.e., when varying latent space size, loss function, or in‐field metric used for adaptation). MSE is a per‐pixel loss function; therefore, it is expected to be quite sensitive to minimal variations or errors. The VAE loss contains the second regularizer term that has the effect of keeping similar (nominal) image representations close together in the latent space, whereas penalizing representations of unseen images that are more likely to cause failures. Moreover, we investigate whether the output of the misbehavior predictors is characterized by different levels of blurriness. To quantify blur, we compute the Laplacian variance for each image.^24^ The Laplacian variance increases with an increased focus of an image or decreases with increased blur. Hence, images with a smaller amount of edges tend to have a smaller Laplacian variance (the Laplacian kernel is often used for edge detection in images).

### 5.4 Results

#### 5.4.1 Results for RQ_1_ (LFP reduction)

Table 2 presents the results for the first study about the reduction of the false positives in nominal conditions.

**TABLE 2:** RQ_1_: Likely false positive reduction at TPR95 for all misbehavior predictors, for both in‐field confidence metrics

Model		VAE	RDR/CWR (MC‐Dropout)			RDR/CWR (CTE)		
(lat. dim./loss)	Dataset	LFP *↓*	LFP *↓*	LFP red. % *↑*	$\bar{CF}\;↓$	LFP *↓*	LFP red. % *↑*	$\bar{CF}\;↓$
2/MSE	Lake	167	67/59	60/65	39/24	65 / 59	61/65	45/27
	Jungle	178	111/90	38/49	109/58	120/119	32/33	138/92
	Mountain	124	69/41	44/67	38/34	17/3	86/98	54/46
	All	156	82/63	47/60	62/**39**	67/60	60/**65**	79/**55**
2/VAE	Lake	165	110/52	33/68	48/21	62 / 59	62/64	36/19
	Jungle	174	105/75	40/57	71/68	50/48	71/72	120/86
	Mountain	91	114/88	−25/3	44/39	90/67	1/26	44/44
	All	143	110/72	16/43	54/43	67/58	45/54	67/50
4/MSE	Lake	127	6/5	95/96	46/51	40/23	69/82	42 / 42
	Jungle	183	90/64	51/65	51/36	58/27	68/85	70/47
	Mountain	146	24/12	84/92	54/46	27/26	81 / 82	56/44
	All	152	40/27	77/84	50/44	42/25	73/83	56/44
4/VAE	Lake	150	49/21	67/86	40/26	42/37	72/75	42/21
	Jungle	129	56/54	57/58	35/29	27/14	79/89	69/51
	Mountain	108	35/11	68/90	36/28	14/11	87/90	28/29
	All	129	47/29	64/**78**	37/**28**	28/21	79/**85**	47/**34**
8/MSE	Lake	125	12/1	90/99	42/57	36/15	71/88	46/40
	Jungle	188	39/37	79/80	62/39	15/4	92/98	87/59
	Mountain	174	21/3	88/98	54/50	8 / 0	95/100	56/48
	All	162	24/14	86/93	53/49	20/6	86/95	61/51
8/VAE	Lake	135	25/7	81/95	31 / 23	33/30	76/78	31/21
	Jungle	201	44/25	78/88	54 / 38	4/2	98/99	66/55
	Mountain	128	5/1	96/99	43/37	3 / 0	98/100	46/37
	All	155	25/11	85/**94**	43/**33**	13/11	90/**92**	48/**38**
16/MSE	Lake	148	5/0	97/100	41/43	31/16	79/89	41/37
	Jungle	201	50/44	75/78	57/42	6/2	97/99	63/55
	Mountain	114	8/5	93/96	34/32	11/5	90/96	38/32
	All	154	21/16	88/91	44/39	16/8	89/95	48/41
16/VAE	Lake	142	5/1	96/99	32/31	28/8	80/94	35/27
	Jungle	190	32/30	83/84	43/30	0/0	100/100	50/40
	Mountain	116	4/2	97/98	40/34	0/0	100/100	42/35
	All	149	14/11	92/**94**	38/**32**	9/3	93/**98**	42/**34**

*Note*: *↓* indicates that lower values are better; *↑* indicates that higher values are better. Best combinations of LFP red. % *↑* and $\bar{CF}$ over all tracks are highlighted in bold and gray color on a per latent dimension basis (2, 4, 8, 16).

Abbreviations: CTE, cross‐track error; CWR, confidence‐based weighted retraining; MC, Monte Carlo; MSE, mean squared error; RDR, rebalanced dataset retraining; VAE, variational autoencoder.

For each VAE configuration being considered, and for each in‐field confidence metric (MC‐Dropout and CTE), the table shows the considered dataset, the number of likely false positives (LFP) by the VAE adopted in SelfOracle, the number of likely false positives (LFP) detected by the considered configuration, both numerically and percentage‐wise, and the CF metric ( $\bar{CF}$). Averages across all tracks are also reported for each configuration. We considered a confidence threshold of ϵ=0.05 (i.e., at TPR = 95%). We recall that in nominal conditions, the ideal situation for a misbehavior predictor would be to have FPR = 0 and TNR = 1. A false positive represents a false alarm by our framework, whereas true negative cases occur when our framework signals correctly the detection of a normal condition.

Our results show that the baseline VAE experiences a large number of false positives for any track (more than a hundred, on average), confirming the results of a previous study.^15^ In practice, this means that the misbehavior predictor equipped with the baseline VAE would raise a high number of erroneous warnings to the main driving component, or to the human driver, even in nominal conditions.

On the other hand, both our proposed techniques are capable to reduce the impact of such false alarms to a large extent, showing high debiasing effects for inherent latent class imbalances. For both RDR and CWR, the decrease is higher for latent spaces with a size higher than 2.

CWR performs constantly better or equal than RDR, rating a higher percentage reduction of LFP (LFP red. %) and a lower CF for all configurations. For several configurations (16/MSE CWR on Lake track, 8/MSE CWR on Mountain track, 8/VAE on Mountain track, 16/VAE on Jungle and Mountain tracks) the number of false alarms is reduced to zero. Statistical significance of the difference was confirmed by applying the nonparametric Mann–Whitney *U* test,^25^ with a confidence threshold α=0.05, with a small Cohen's *d* effect size.^26^ About VAE versus CWR, the LFP reduction differences were found statistically significant with a large effect size. About RDR versus CWR, the differences were found statistically significant with a small effect size for LFP reduction.

Moreover, concerning CF, we can notice that CWR induces a lower reconstruction error deterioration on the driving scenes that were reconstructed well (i.e., whose reconstruction error was already below the 95% threshold after the first training), highlighted by equal to lower $\bar{CF}$ values than RDR across all configurations and for both confidence metrics. The differences were found statistically significant with a medium effect size. In conclusion, this means that a large number of true negatives are confirmed after weighted retraining with CWR, and a substantial portion of false positives become true negatives (Figure 7).

Looking at the individual tracks, for the Lake track, the reduction is higher with increasing sizes of the latent space (for 16/MSE, we have 100% reduction). For the Jungle track, predictors with a latent space size of 8 work best when considering both MC‐Dropout or CTE. For the Mountain track, both dimensions 8 and 16 are equally good, especially with CTE, whereas a low dimension of 2 experienced the worst performance in our study. For the MC‐Dropout metric, the predictors equipped with VAE loss were found more effective for latent space dimension bigger than 4. For the CTE metric, the predictors equipped with MSE loss were found more effective for latent space dimension equal to 2 and 8.

**RQ**_1_: *The misbehavior predictors retrained using confidence‐driven weighted retraining (CWR) reduce the LFP rate up by a large margin (up to zero in some configurations). Therefore, confidence‐driven weighted retraining offers clear advantages over rebalanced dataset retraining (RDR) both in terms of LFP reduction rate and training cost (training size and time)*.

#### 5.4.2 Results for RQ_2_ (misbehavior prediction)

Table 3 shows the results for the misbehavior prediction in unseen conditions. For each VAE model configuration being considered, and for each in‐field confidence metric (MC‐Dropout and CTE), the table shows the considered dataset, the precision, recall, and F‐1 scores at FPR95. We reported the results for an TTM = 3, which means having a detection and reaction window of 3 s prior to the occurrence of the misbehavior. Moreover, we report the AUC‐ROC score, as well as the AUC‐PRC score.

**TABLE 3:** RQ_2_: Misbehavior prediction results for all misbehavior predictors

Model		VAE					RDR/CWR (MC‐Dropout)					RDR/CWR (CTE)				
(lat. dim./loss)	Dataset	Prec. (%)	Rec. (%)	F‐1 (%)	AUC‐ROC	AUC‐PRC	Prec. (%)	Rec. (%)	F‐1 (%)	AUC‐ROC	AUC‐PRC	Prec. (%)	Rec. (%)	F‐1 (%)	AUC‐ROC	AUC‐PRC
2/MSE	Lake	60	100	75	0.97	0.80	82/75	100/100	90/86	0.99/0.99	0.91/0.88	70/82	100/100	82/90	0.98/0.99	0.85/0.91
	Jungle	56	36	43	0.66	0.50	81/66	36/32	50/43	0.67/0.65	0.63/0.53	74/100	36/28	49/44	0.67/0.64	0.59/0.69
	Mountain	64	61	62	0.78	0.65	52/78	42/68	47/73	0.69/0.83	0.50/0.75	86/74	79/74	82/74	0.89/0.85	0.84/0.75
	All	60	66	60	0.80	0.65	72/73	59/67	62/**67**	0.78/0.82	0.68/0.72	77/85	67/72	69/**71**	0.85/0.83	0.76/0.78
2/VAE	Lake	57	100	72	0.96	0.78	70/82	100/100	82/90	0.98/0.99	0.85/0.91	75/75	100/100	86/86	0.99/0.99	0.88/0.88
	Jungle	53	27	36	0.62	0.44	66/81	32/36	43/50	0.65/0.67	0.53/0.63	91/85	44/48	59/61	0.72/0.73	0.71/0.70
	Mountain	38	18	24	0.60	0.38	35/35	21/21	26/26	0.58/0.58	0.33/0.33	45/39	26/21	33/27	0.61/0.59	0.40/0.35
	All	49	48	44	0.73	0.53	57/66	51/52	50/55	0.74/0.75	0.57/0.62	70/67	57/56	59/58	0.77/0.77	0.66/0.64
4/MSE	Lake	60	100	75	0.97	0.80	100/90	100/100	100/95	1.00/1.00	1.00/0.95	82/82	100/100	90/90	0.99/0.99	0.91/0.91
	Jungle	75	59	66	0.78	0.70	76/100	68/72	72/84	0.82/0.86	0.74/0.88	81/100	72/72	76/84	0.85/0.86	0.78/0.88
	Mountain	71	83	76	0.89	0.78	69/69	100/100	82 / 82	0.97/0.97	0.84/0.84	66/69	100/100	79/82	0.97/0.97	0.83/0.84
	All	69	81	72	0.88	0.76	82/86	89/91	85/**87**	0.93/0.94	0.86/0.89	76/84	91/91	82/**85**	0.94/0.94	0.84/0.88
4/VAE	Lake	53	100	69	0.96	0.76	70/75	100/100	82/86	0.98/0.99	0.85/0.88	65/75	100/100	79/86	0.98/0.99	0.82/0.88
	Jungle	85	59	69	0.79	0.78	86/93	52/56	65/70	0.75/0.78	0.72/0.77	100/93	52/56	68/70	0.76/0.78	0.79/0.77
	Mountain	69	61	65	0.78	0.67	82/87	90/84	86/85	0.94/0.91	0.86/0.86	87/93	90/90	88/91	0.94/0.94	0.89/0.92
	All	69	73	68	0.84	0.74	79/85	81/80	78/80	0.89/0.89	0.81/0.84	84/87	81/82	78/82	0.89/0.90	0.83/0.86
8/MSE	Lake	60	100	75	0.97	0.80	82/75	100/100	90/86	0.99/0.99	0.91/0.88	90/75	100/100	95/86	1.00/0.99	0.95/0.88
	Jungle	69	63	66	0.80	0.68	95/100	84/84	89/91	0.92/0.92	0.91/0.93	100/95	80/84	89/89	0.90/0.92	0.91/0.91
	Mountain	76	86	80	0.96	0.91	63/72	100/100	77/84	0.96/0.98	0.82/0.86	69/69	100/100	82/82	0.97/0.97	0.84/0.84
	All	68	83	74	0.91	0.80	80/82	95/95	86/87	0.96/0.96	0.88/0.89	86/80	93/95	88/86	0.96/0.96	0.90/0.88
8/VAE	Lake	57	100	72	0.96	0.78	70/75	100/100	86/82	0.98/0.99	0.85/0.88	65/75	100/100	79/86	0.98/0.99	0.82/0.88
	Jungle	78	68	72	0.82	0.75	95/100	76/80	84/89	0.88/0.90	0.87/0.91	95/100	80/80	87/89	0.90/0.90	0.89/0.91
	Mountain	66	66	66	0.81	0.68	82/100	90/95	86/97	0.94/0.97	0.86/0.98	100/100	90/95	94/97	0.95/0.97	0.95/0.98
	All	67	78	70	0.86	0.74	82/92	89/92	85/**89**	0.93/0.95	0.86/0.92	87/92	90/92	87/**91**	0.94/0.95	0.89/0.92
16/MSE	Lake	57	100	72	0.96	0.78	100/75	100/100	100/86	1.00/0.99	1.00/0.88	90/75	100/100	95/86	1.00/0.99	0.95/0.88
	Jungle	76	77	77	0.87	0.78	95/100	84/84	89/91	0.92/0.92	0.91/0.93	100/100	84/84	91/91	0.92/0.92	0.93/0.93
	Mountain	76	86	80	0.96	0.91	66/72	100/100	79/84	0.97/0.98	0.83/0.86	66/69	100/100	79/82	0.97/0.97	0.83/0.84
	All	70	88	76	0.93	0.82	87/82	95/95	90/87	0.96/0.96	0.91/0.89	85/81	95/95	89/86	0.96/0.96	0.90/0.88
16/VAE	Lake	57	100	72	0.96	0.78	65/70	100/100	79/82	0.98/0.98	0.82/0.85	70/70	100/100	82/82	0.98/0.98	0.85/0.85
	Jungle	71	59	65	0.78	0.67	100/100	80/84	89/91	0.90/0.92	0.91/0.93	100/100	80/84	89/91	0.90/0.92	0.91/0.93
	Mountain	81	61	70	0.79	0.73	100/100	90/90	94/94	0.95/0.95	0.95/0.95	100/100	90/90	94/94	0.95/0.95	0.95/0.95
	All	70	73	69	0.84	0.73	88/90	90/91	87/**89**	0.94/0.95	0.90/0.91	90/90	90/91	88/**89**	0.94/0.95	0.90/0.91

*Note*: Best combinations of F‐1 over All tracks are highlighted in bold and gray color on a per latent dimension basis (2, 4, 8, 16). Scores are computed at a FPR95 and TTM = 3 s.

Abbreviations: AUC‐PRC, area under the precision–recall curve; AUC‐ROC, area under the curve of the receiver operating characteristics; CTE, cross‐track error; CWR, confidence‐based weighted retraining; MC, Monte Carlo; MSE, mean squared error; RDR, rebalanced dataset retraining; VAE, variational autoencoder.

Results of our retrained misbehavior predictors confirm that most true alarms are detected correctly after adaptation. Apart from a few configurations, the recall and precision values are generally high for all predictors with latent space sizes greater or equal to 4. F‐1, AUC‐ROC, and AUC‐PRC are also high for predictors with latent space size greater or equal to 8. The original unretrained VAE misbehavior predictor obtains good prediction results, confirming previous results.^15^ However, the effectiveness is lower than our proposed technique CWR for either of the two variants MC‐Dropout and CTE, with statistical significance measured by the nonparametric Mann–Whitney *U* test,^25^ with a confidence threshold α=0.05, with a medium to large Cohen's *d* effect size,^26^ respectively.

Concerning the in‐field confidence metric, both tool configurations show competitive results for both MC‐Dropout and CTE. Indeed, no large differences were expected in terms of misbehavior prediction effectiveness, as both tool configurations share the same VAE architecture and training set, with the only difference being the way in which they are trained. Indeed, statistical analysis also revealed no statistically significant differences. However, it is important to remark that the RDR technique has a major drawback, as it requires a non‐negligible overhead in terms of additional training set images to be added to the original training set (on average, directly proportional to the number of images being reconstructed badly). On the contrary, CWR requires no training set image to be added, and it works by just reweighing the input samples according to the observed in‐field confidence metrics.

Concerning the TTM, we found empirically that no major differences were observed in a time window between 1 and 3 s far from the misbehaviors, on average. Conversely, when the considered time window increased over 4 s, the prediction capability decreases. Our own experience with the Udacity simulator confirms that a detection and reaction time of 3 s is sufficient to anticipate most misbehaviors at a speed of 30 mph.

**RQ**_2_: *The misbehavior predictors retrained using confidence‐driven weighted retraining (CWR) exhibit a high misbehavior prediction rate. Therefore, confidence‐driven weighted retraining attains a high TPR rate on unseen conditions allowing anticipating a large number of misbehaviors (up to 100% in some configurations) up to to 3 s ahead*.

#### 5.4.3 Results for RQ_3_ (internal validation)

##### Results for RQ_3.1_ (loss)

Concerning the LFP reduction, we consider misbehavior predictor configurations with the same latent space size. In the case of MC‐Dropout, CWR's VAE configurations show an LFP reduction for latent dimensions greater or equal to 8. In the case of CTE, all CWR's VAE configurations are better than MSE in terms of LFP reduction.

**FIGURE 8:**
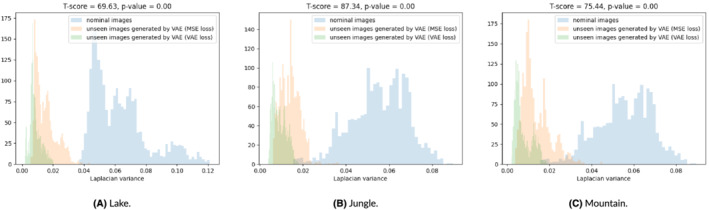
Laplacian variance of the reconstructed nominal/unseen driving images by our best misbehavior predictor. (A) Lake, (B) Jungle, and (C) Mountain

Concerning the misbehavior prediction, we compare the AUC‐ROC scores across misbehavior predictor configurations with the same latent space size. In the case of MC‐Dropout, CWR's MSE configurations show better AUC‐ROC scores for all latent dimensions. The same result is confirmed also for the CTE metric. Images generated by the misbehavior predictors using the VAE loss tend to be more blurry than images generated by misbehavior predictors using the MSE loss for the same images, according to the Laplacian variance (Figure 8).

On average, the nominal images have the highest Laplacian variance (highest focus or least blur), whereas the reconstructed images are more blurry. The images reconstructed by predictors with MSE loss are significantly less blurry than those reconstructed by those using the VAE loss. The statistical significance of this difference was assessed with a *t* test for paired samples^27^ (i.e., the same test images are used by both AEs). The *t* score is quite large and the *p* value is zero, definitely lower than 0.05. We can therefore conclude that images generated by the predictors with the MSE loss are significantly less blurry than those generated by the predictors with the VAE loss. The lower blur level associated with MSE results in reduced false alarms (see LFP in Table 2) and accurate misbehavior prediction (see F‐1 in Table 3).

**RQ**_3.1_: *The misbehavior predictors using the MSE loss function showed superior performance both in reducing the LFP rate as well as in accurately predicting misbehaviors*.

##### Results for RQ_3.2_ (latent space size)

We compare the LFP reduction across misbehavior predictor configurations with the same latent space size. In the case of MC‐Dropout, all configurations show a significant LFP reduction when the dimension increases. Indeed, the best results are obtained by the predictors with latent space equal to 16. The same result is confirmed also for the CTE metric.

Concerning the misbehavior prediction, we compare the AUC‐ROC scores across misbehavior predictor configurations with the same latent space size. In the case of MC‐Dropout, all configurations show a high AUC‐ROC score when the dimension is greater or equal to 8. Indeed, the best results are obtained by the predictors with latent space equal to 16. The same result is confirmed also for the CTE metric.

**RQ**_3.2_: *In our autonomous driving domain, the misbehavior predictors using a bigger latent dimension (16, in our experiments) showed superior performance both in reducing the LFP rate as well as in accurately predicting misbehaviors*.

##### Results for RQ_3.3_ (in‐field confidence metric)

Table 4 shows the LFP detection rate for MC‐Dropout and CTE across all configurations on a per‐track level. Averages across tracks are also reported. Overall, both metrics exhibited a high detection rate (over 90%), according to the ruleset of Table 1. The accurate detection of in‐field nominal driving conditions benefited our framework to a large extent (see results for RQ_1_ and RQ_2_).

**TABLE 4:** Likely false positive detection by in‐field confidence metric

	Lake					Jungle					Mountain					All tracks				
Model		MC‐Drop.		CTE			MC‐Drop.		CTE			MC‐Drop.		CTE			MC‐Drop.		CTE	
(lat. dim./loss)	LFP	#	%	#	%	LFP	#	%	#	%	LFP	#	%	#	%	LFP	#	%	#	%
2/MSE	167	146	87	113	78	178	174	98	178	100	124	91	73	124	100	469	411	88	415	88
2/VAE	165	152	92	114	89	174	174	100	174	100	91	72	79	91	100	430	398	93	379	88
4/MSE	127	113	89	86	78	183	183	100	183	100	146	142	97	146	100	456	438	96	415	91
4/VAE	150	136	91	107	81	129	129	100	129	100	108	87	81	108	100	387	352	91	344	89
8/MSE	125	116	93	89	81	188	174	93	185	98	174	167	96	174	100	487	457	94	448	92
8/VAE	135	124	92	101	85	201	186	93	201	100	128	118	92	128	100	464	428	92	430	93
16/MSE	148	138	93	114	87	201	183	91	198	99	114	105	92	114	100	463	426	92	426	92
16/VAE	142	132	93	112	89	190	176	93	190	100	116	106	91	116	100	448	414	92	418	93
Average	145	132	**91**	105	**84**	181	172	**96**	180	**100**	125	111	**88**	125	**100**	451	416	**92**	409	**91**

Abbreviations: CTE, cross‐track error; MC, Monte Carlo; MSE, mean squared error; VAE, variational autoencoder.

Looking at the results for each individual track, for Lake track, MC‐Dropout detected 27 LFP more than CTE (+26%). CTE is an approximated measure (see Section 4.1.1), and the approximation error is larger on long bends, which necessitates a larger number of waypoints to approximate the central trajectory. The DAVE‐2 model can occasionally drive quite far from such a reference trajectory, without necessarily exhibiting major misbehaviors. In fact, the sequence of steering angles to face a 90° bend differs depending on the speed at which the vehicle approaches the bend. DAVE‐2 is indeed able to generalize quite well to our tracks also to speeds and conditions that differ from the exact driving scenes captured in the training set. In such a situation, MC‐Dropout may be better at estimating the confidence of the driving component.

On the contrary, for the Mountain track, CTE offers a better confidence estimation because the ideal trajectory is easier to approximate with waypoints. The road section of the track allows for multiple correct trajectories to be taken to face the long gentle bends of the track. Indeed, MC‐Dropout lost a few frames (14, amounting to −13%) in which the car deviates substantially from the ideal trajectory. Such frames, however, were correctly captured by CTE.

For the Jungle track, both metrics score very high detection rates. MC‐Dropout lost a few frames (9, −5%) because, unlike Mountain track, the road section is quite narrow, and correspondingly, the car may suddenly face a hazardous condition. The MC‐Dropout metric failed to immediately report a decrease in the confidence level of DAVE‐2, whereas the CTE metric correctly detected it.

**RQ**_3.3_: *Both MC‐Dropout and CTE offer competitive results as in‐field confidence metrics. In scenarios where it is possible to measure CTE accurately, we suggest to use it as the reference confidence metric for an autonomous driving model that performs lane‐keeping*.

### 5.5 Threats to validity

We compared all variants of our framework and our baseline VAE under the same evaluation sets and parameter settings. A threat to internal validity concerns the training of self‐driving car models, which may exhibit a large number of misbehaviors if trained inadequately. We mitigated this threat by training and fine‐tuning the best publicly available driving models. Our custom implementation of the MC‐Dropout and CTE metrics within the Udacity simulator constitutes another threat to validity that we mitigated by testing our implementation extensively.

In terms of generalization of our results, we rely on the Udacity simulator's capabilities to reflect the autonomous driving model in the real‐world, and our data might not generalize to different simulation platforms. Datasets of real driving scenes cannot be used in our study because we cannot compute our metrics and observe failures for such driving scenarios.

All our results, the source code, the simulator, and all subjects are available,^28^ making the evaluation repeatable and our results reproducible.

## 6 DISCUSSION

### 6.1 Analysis of the results

Our evaluation revealed that the DAVE‐2 self‐driving car model generalizes differently from the VAE‐based misbehavior predictor in case of class imbalance. The misbehavior predictors based on the VAE, on the other hand, are not likewise able to learn meaningful nominal patterns if class imbalance affects the training set. The latent space of a VAE trained on uniformly weighted data contains many poor‐performing samples that cause many false positives in practice when used in the field.

Weighted retraining mitigates such a problem by introducing more high‐performing points into the latent space using a combination of confidence‐driven sample selection and AE loss‐based weight initialization. Weighted retraining robustly balances the distribution between poorly reconstructed samples at the expense of good reconstructed values, which is the intended effect. The gist is that the results on both minimizations of false alarms and misbehavior prediction are broadly supportive of our approach, which improves an existing state‐of‐the‐art AE‐based misbehavior predictor for autonomous driving systems.

Our results also show that it is possible to use MC‐Dropout to obtain reliable uncertainty scores, confirming previous studies.^23^ This might suggest that it would be possible to build misbehavior predictors relying solely on white‐box metrics. However, such results should be taken with care because MC‐Dropout predictions can be computationally demanding for an online setting. Even if we do not report extensive performance results, the performance of the main driving component and the overall driving quality were not affected if the number of samples is kept the same as the batch size (i.e., 128 in our experimental setting), making our framework a real‐time viable solution. Conversely, the driving quality was affected when increasing the batch size value is increased. Thus, MC‐Dropout is a valid choice for online runtime drift detection, especially if accelerator‐specific hardware is available within the deployed system (e.g., tensor processing units).

Even if CTE is commonly used in domains such as aircraft landing systems,^29^ in the self‐driving car domain, it is not a standard metric yet as it may not always be available, because it requires the capability to compare the ideal with the actual trajectory. Current industrial self‐driving cars can accurately approximate metrics such as CTE by means of advanced computer vision algorithms or dedicated sensors. In the future, CTE may become more standard and utilized. The advent of smart cities and dedicated roadways designed for autonomous vehicles could provide online information about the car's driven lane position. CTE is also increasingly being utilized within reward functions of self‐driving cars based on reinforcement learning algorithms.^30^

The best configuration from our experiments is the misbehavior predictor paired with MC‐Dropout as drift detector, the VAE loss function, and a latent space of 16. This essentially means that a latent vector of size 2 as initially used in the paper by Stocco et al^15^ is too constrained to hold all meaningful differences for the images of the training set. However, a careful selection of the latent space dimension is necessarily domain‐dependent. Concerning the in‐field confidence metrics used by our framework, both MC‐Dropout and CTE contributed to a competitive reduction of the likely false‐positive rate. When available, CTE might be a good choice in resource‐constrained settings, where the available hardware is not compatible with the expensive online uncertainty computation performed by MC‐Dropout.

### 6.2 Applications

Our monitoring framework applies to both fail‐passive and fail‐active systems. In the former case, it can be used to prevent failures from happening by promptly warning the driver or the main driving component about an unsupported driving scenario. In the latter case, it can be used as a component within a fail‐safe mechanism that tries to react in a way that minimal harm is caused to the vehicle, to the environment or to the people.

In this work, we consider black‐box environmental monitors, specifically VAEs. However, our adaptation framework is quite generic and can be applied to any machine learning‐based technique used to monitor the environment and identify anomalies from imagery data. The main working assumption is to use a scoring function that allows accurate differentiation between nominal and anomalous images.

The data mined from the field can be also used for the retraining of the main system. However, such data lack labels, which in practice are costly to get as labeling requires human effort. In the case of a self‐driving car, this problem is even more difficult because it is challenging if not impossible to manually assign a meaningful label (i.e., a steering angle) only by looking at individual images. A more accurate but expensive option would require a human driver to drive in a simulated environment as close as possible to the parts of the track in which the likely false positives were detected. The control actions (e.g., steering angles) performed by the human would then serve as ground truth labels. Automated test generation techniques could be used to produce a simulation environment that mimics the underrepresented conditions found in the field.^20^

For underrepresented frames, an approximate label could be found by looking at the most similar image in the training set, which can be compared with the one by the autopilot. A *k*‐nearest neighbor search based on some image similarity metric such as the SSIM^31^ can be adopted to search the training set for samples that are close to the ones collected in the field.

We considered a setup in which negative examples are unknown; thus, they cannot be used at training time. If knowledge of the expected true anomaly rate applies to the context, there are multiple options that can be explored. First, knowledge of anomalies can be used to select a more precise threshold. Another direction would be to retrain more accurate monitors over time using sophisticated AE architectures. Triplet loss function within AEs^32^ can minimize the distance (maximize the similarity) between in‐distribution and positive samples, while maximizing the distance (minimizing the similarity) between in‐distribution and negative samples. Adversarial AEs^33^ adopt two networks with competing objectives: A generator attempts to reconstruct images that belong to the nominal data distribution, whereas a discriminator tries to distinguish whether the samples belong to the nominal or to the anomalous data distribution.

We are confident that our framework can also support unseen scenarios similar to the ones used in our experiment (i.e., snow and fog) because they are expected to perturb the camera images used for steering angle prediction analogously to rain, perhaps with different levels of magnitude. Other semantically similar image perturbations would consist in perturbing a ratio of images along the stream to simulate malfunctions of the main camera. Generalization of our results to such unseen scenarios is left for future work.

Concerning other future improvements, one might study other predictive uncertainty metrics that take epistemic uncertainty into account (which is what occurs during data distribution shift) and are efficient for real‐time online settings. A promising option is given by temperature scaling,^34^ whereas ensembles of models, despite being regarded as the best solution,^35^ are not expected to scale well to real‐time scenarios due to their high computational cost.

### 6.3 Open challenges

One major challenge concerns the deployment of new retrained monitors without negatively affecting the main system's behavior. Moreover, it is important to strike a balance between old and new knowledge by maintaining also some samples of older data distribution within an archive, to avoid overfitting the data distribution towards only the most recent data that is collected at runtime. To this extent, generative replay by the encoder's part of AEs has shown promising results.^36^

Techniques should be also devised to maintain the training set size limited only to the most representative examples, as the retraining time scales up with the size of the training set. Training set minimization techniques can be useful in this context such as those based on stratified sampling or Synthetic Minority Oversampling Technique (SMOTE).^37^ Also, different misbehaviors may have a different TTM window sizes, whereas in our experiments, we used a fixed window size.

Another open challenge concerns the coupling between the main system and the monitor. The misbehavior predictor's effectiveness may diminish in cases whereby the main system produces reasonable in‐field behavior also for inputs that should be regarded as anomalies. Thus, including such inputs in the anomaly detector's training set might introduce a drift in the monitor's knowledge, which may become less sensitive to behaviors similar to the undetected anomalous scenarios. In our experiments, this situation did not occur, as most system‐level failures were correctly detected by the monitor both pre‐ and post‐adaptation. However, the coupling between the main system and the safety monitor may have far‐reaching consequences that require further investigation.

Lastly, another possible line of research consists of studying the automated generation of countermeasures upon detection of an hazard, such as reducing the speed, braking, or parking the vehicle into the nearest safe location or combinations of those. This healing component must be designed carefully as false alarms are not only uncomfortable, but they could also be unsafe.

Our system is similar to the safety systems already in place in existing vehicles such as those for emergency braking. Such systems try to prevent rear‐end collisions or mitigate their consequences by using sensors to assess whether a collision is likely. The system will usually start by warning the driver, using a dashboard message or an audible alarm. If the driver fails to take action, the automatic or “autonomous” part of the system will apply the brakes automatically.

In our work, we focus on the early detection of potentially unsupported driving scenarios by the autopilot so that either the human driver or the main driver component can be warned promptly. We do not focus on what actions are best for a particular driving scene, which is a possible follow‐up of our work.

## 7 RELATED WORK

In this section, we overview the main related work in the autonomous vehicle testing domain, along the following dimensions: (1) monitoring systems, (2) quality assessment, and (3) test generation.

### 7.1 Monitoring systems for autonomous driving systems

Concerning DNN‐based system monitors for autonomous vehicles, Henriksson et al^38^ use the negative of the log‐likelihood of the images generated by a VAE as an anomaly score for driving images. However, the data distribution of the images in their test set (urban scenes) is by far quite different from that of the training set (highway scenes). In our experiment, we create the anomalous set by gradually injecting anomalous conditions starting from the training set tracks, which allows a more realistic transition from nominal to unexpected scenarios. Strickland et al^39^ use a convolutional LSTM embedding multiple indicators such as the image of the driving environment and the state of the vehicle to predict impeding collisions with other vehicles at crossroads. Michelmore et al^23^ proposed the use of MC‐Dropout to estimate the confidence of an autonomous vehicle and use it to predict unsafe situations. In a follow‐up work, Michelmore et al^40^ use several Bayesian inference methods and show that uncertainty estimates are highly effective to support decision making in autonomous driving. Langford and Cheng^41^ propose Enki to generate unsupported environmental conditions that expose misbehaviors for a DNN trained for the CIFAR‐10 benchmark. In a recent work, Langford and Cheng^42^ combine evolutionary computation with machine learning to produce a method to predict misbehaviors of a DNN‐based object detector for autonomous driving when faced with previously unseen environmental conditions. Differently, in this paper, we use both white‐box and black‐box confidence metrics for improving the effectiveness of a monitor based on AEs that are improved through weighted retraining.

### 7.2 Quality assessment for autonomous driving systems

Concerning quality metrics for evaluating the driving performance, Jahangirova et al^21^ evaluated 26 metrics related to the quality of driving of both human and autonomous driving and showed their usefulness as functional oracles through mutation testing.^43^ Evans et al^44^ propose the design of a domain‐specific language to express oracles for autonomous driving systems testing. Such a language, ideally, would allow encoding safety, liveness, timeliness, and temporal properties. Ayerdi et al^45^ propose a taxonomy to elicit requirements for Design‐Operation Continuum Engineering of CPS. The taxonomy has been applied to the elevation and railway domains; applicability and usefulness to autonomous driving have to yet be verified.

Researchers have also investigated the relation between online and offline metrics. In the lane keeping task, offline metrics refer to the error associated with the prediction of the ground‐truth steering angle, whereas online metrics refer to misbehaviors occurring during a simulation, such as collisions. Codevilla et al^46^ found that offline prediction errors are not correlated with driving quality, and two models with comparable error prediction rates may differ substantially in their driving quality. Similarly to Codevilla et al.,^46^ Haq et al^47^ performed an empirical study comparing offline and online testing of DNNs for autonomous driving. The goal was to understand whether simulator‐generated data can be a reliable proxy for real‐world data. Results show that simulator‐generated data yield similar prediction errors as those obtained on real‐world datasets. Moreover, offline testing is less viable in exposing safety violations than online testing. Specifically, all severe violations exposed by simulations are also exposed by offline techniques/measures, but the opposite is not true. The way authors compare simulated with real data is by generating a high number of scenarios in the simulator until sequences of images that have analogous labels (i.e., steering angles) are found. Differently, in this paper, we use driving quality metrics such as the CTE as confidence values for the retraining of a system monitor that aims to predict misbehaviors.

### 7.3 Test generation for autonomous driving systems

Test generation techniques for self‐driving cars aim at automatically constructing test cases for vision‐based autopilots.^48^, ^49^, ^50^, ^51^, ^52^, ^53^, ^54^ Test cases are represented by images of driving scenes as seen by a human driver or images that represent road shapes that are rendered within a simulation platform. Abdessalem et al^48^, ^49^, ^50^ combine genetic algorithms and machine learning to test a pedestrian detection system. Mullins et al^55^ use Gaussian processes to drive the search towards yet unexplored regions of the input space, whereas Gambi et al^56^ propose asfault, a search‐based test generator for autonomous vehicles based on procedural content generation. asfault uses search operators that mutate and recombine road segments to construct road networks for testing the lane keeping functionality of self‐driving cars. Riccio and Tonella^20^ propose DeepJanus, a model‐based test generator that uses Catmull–Rom splines to characterize the road shape and generate inputs that are at the behavioral frontier of a self‐driving car model. Zohdinasab et al^57^ use illumination search to cover the map of external behaviors of a self‐driving vehicle. Riccio et al^58^ augment existing test suites by mutation adequacy‐guided test generation. Arrieta et al^59^ use a genetic algorithm to generate tests for CPS that optimize along three directions, namely, requirement coverage, test case similarity, and test execution time. The goal of test generators is to derive extreme and challenging roads, maximizing the number of observed failures, while our goal is to predict system‐level failures in online mode. In contrast to the existing works, we study specifically how to adapt a VAE‐based anomaly detector in the self‐driving car domain, using in‐field confidence metrics (predictive uncertainty and lateral deviation) as drift detectors. To the best of our knowledge, our study is the first that combines continual learning with in‐field metrics, such as predictive uncertainty, to detect distribution drifts of the input data and to drive the retraining of a better VAE. We carried out a comparison with the online misbehavior prediction of SelfOracle,^15^ finding poor performance of the VAE in presence of data distribution shifts.

Other works propose adversarial input generation to generate inputs that trigger inconsistencies between multiple autonomous driving systems^51^ or between the original and transformed driving scenarios.^52^, ^53^, ^60^ For example, DeepExplore,^51^ DeepTest,^52^ and DeepRoad^53^ test the robustness of self‐driving car modules. In these papers, the proposed frameworks synthesize alternative driving images simulating adversarial driving conditions. DeepExplore^51^ uses lightning effects and occlusions to transform the testing data into artificially simulated adversarial inputs. DeepTest^52^ alters the images using synthetic affine transformations from the computer vision domain, such as blurring and brightness adjustments, and Photoshop to create simulated rain/fog effects. Differently, DeepRoad^53^ generates images by means of Generative Adversarial Networks (GANs). Generated images are then validated by measuring their distance in the latent space produced by PCA with respect to the training images. Results show that unseen scenarios created in this way have larger MSE; therefore in many cases, they fool the DNN that predicts the steering angle. Such solutions alter the input images fed to the DNN by means of artificial transformations (e.g., lightning effects and occlusions^51^ and affine transformations^52^), as well as GANs,^53^ to simulate adversarial driving conditions. However, the main use case concerns the identification of underrepresented scenarios in the training data to support retraining and better generalization after retraining. Indeed, a previous paper highlighted the poor performance of such techniques when used for online misbehavior prediction.^15^ Concerning the definition of unseen driving scenario, the work by Bolte et al^61^ in the autonomous vehicle domain provides a definition of misbehavior based on nonpredictable relevant objects in a relevant location around the car. Such a definition is compatible with our framework, as our reconstruction error component would react to the presence of an anomalous object. Coupled with an object recognition component, we may weigh different parts of the images differently, depending on the presence/absence of an object in them.

## 8 CONCLUSIONS AND FUTURE WORK

Predicting and minimizing safety‐critical system failures in autonomous driving systems is a prerequisite for on‐road deployment. A monitoring system can be helpful, provided that it can quickly adapt to changes in the nominal data distribution. This paper proposes a framework for continual learning of a runtime monitoring system based on a variational AE, which keeps evolving a misbehavior predictor as additional experience of drifting nominal instances becomes available. When the observed instances deviate from the nominal distribution of the data used for training without affecting any driving quality metrics, new samples are collected and incorporated using adaptive weighted retraining. Our experimental results show that both black‐box and white‐box confidence metrics can be used as accurate in‐field drift detectors and that the reduction of the false alarm rate obtained thanks to our technique is substantial. At the same time, the retrained misbehavior predictors attain a high failure prediction capability because our framework is designed to minimize CF upon retraining.

In our future work, we plan to address the remaining challenges, which include the safe deployment of the adapted monitors, the trade‐off between frequent adaptations and introduction of regressions, and the exploitation of knowledge about true anomalies observed in the field.

## Data Availability

The data that support the findings of this study are openly available in GitHub at https://github.com/testingautomated-usi/jsep2021-replication-package-material.

## Footnotes

*
https://www.tesla.com/autopilotAI
